# Seeing Beyond the Human Eye: The Video Laryngoscope as the Anesthesiologist’s Backup Camera

**DOI:** 10.7759/cureus.93421

**Published:** 2025-09-28

**Authors:** Marcos A Lessa, Camila A Soriano

**Affiliations:** 1 Department of Anesthesia, University of Iowa Hospitals and Clinics, Iowa City, USA; 2 Department of Anesthesia, Brazilian National Institute of Cancer, Rio de Janeiro, BRA

**Keywords:** airways, procedure safety, skills and simulation training, technology enhanced education, video-laryngoscope

## Abstract

Technological advances have transformed airway management, with the video laryngoscope now widely adopted across anesthetic, emergency, and critical care settings. This editorial draws an analogy between the video laryngoscope and vehicle backup cameras to emphasize the importance of such tools as valuable adjuncts rather than replacements for foundational airway skills. The discussion not only explores how the video laryngoscope enhances glottic visualization and supports safer intubation--especially in scenarios involving limited neck mobility or distorted airway anatomy--but also introduces new challenges, such as the “see but can’t intubate” phenomenon. We examine the pedagogical role of the video laryngoscope in training, its clinical applications across diverse settings, limitations in specific patient populations, and cost-effectiveness considerations. Finally, we reflect on future perspectives, including the integration of artificial intelligence, robotics, and immersive simulation in airway management. While the video laryngoscope represents a major leap forward, its effective use requires continued emphasis on core airway competencies, device-specific training, and thoughtful integration into clinical practice.

## Editorial

Technological evolution in augmenting human visual capacity has led to transformative advances across multiple disciplines. The development of tools facilitating vision beyond innate limitations has catalyzed advances in various scientific fields. Instances include microscopic unveiling of the unseen world in biology and astronomical exploration facilitated by telescopes [1,2]. Similarly, creating tools to enhance visual acuity in specific contexts has profoundly affected daily life and professional pursuits [3,4]. This editorial discusses the parallel advances in two technological innovations: video laryngoscopes and backup cameras.

Development and dissemination

Initially, backup cameras were luxury accessories, but they have become automobile-mandated safety features in the last few years. The evolution of vehicular backup camera technology has progressed from small, low-resolution screens on the dashboard to larger, user-friendly, multicolor screens integrated into the vehicle’s infotainment systems. Contemporary systems also include parking assistance features utilizing 360-degree surround view technology, which combines camera feeds to present a comprehensive bird's eye view of the vehicle's surroundings. The proliferation of backup camera technology is primarily the result of regulatory implementations, such as the U.S. Cameron Gulbransen Kids Transportation Safety Act of 2008 [5]. This legislation was enacted in response to tragic accidents involving rearward vehicle movement and culminated in mandatory rear visibility technology for all new vehicles weighing less than 10,000 pounds by May 2018 [5].

Conversely, video laryngoscopy has transformed from an expensive, specialized instrument to a standard tool in diverse medical settings. The prototype for the video laryngoscope, developed in 1998, was direct laryngoscopy with a fibreoptic attached to the Macintosh blade [6]. Subsequently, other prototypes were created [7], and in 2001, Verathon Medical (Bothell, WA, US) launched the Glidescope®, the first commercially available video laryngoscope [7]. This device integrated a top-quality digital camera and a high-resolution liquid crystal display monitor. Over the years, numerous models featuring varied blade configurations and camera positions have been developed. Specific models even incorporate illumination features, suction capabilities, and procedural documentation capabilities. Prominent medical associations, including the American Society of Anesthesiologists, the Difficult Airway Society, the Association of Anaesthetists, and the Intensive Care Society, have recommended using video laryngoscopy for tracheal intubation in airway management guidelines for years [8-10]. Its widespread use for accessing difficult airways, particularly during the recent COVID-19 pandemic, attests to the value of video laryngoscopes in modern medical practices.

Safety and risk mitigation

Effective and reliable visibility is crucial in both vehicular operations and medical procedures. Backup cameras and video laryngoscopy technologies have revolutionized safety and improved outcomes based on their ability to increase our visual field. Backup cameras have reduced accidents and collisions associated with blind spots, particularly against stationary objects [11]. By offering real-time video feeds of the area directly behind the vehicle and enhanced visibility, backup cameras provide a clear and comprehensive view of the vehicle during reversing maneuvers, reducing the likelihood of backing crashes and increasing driving safety [12]. According to the US National Highway Traffic Safety Administration, backup cameras have the potential to minimize back accidents because they allow drivers to see hazards that are farther away [13]. A study from the Insurance Institute for Highway Safety (IIHS) demonstrated that when paired with parking sensors, backup cameras decrease the area of blind zones by 72-99% [14].

Similarly, video laryngoscopes have transformed anesthesiology and airway management by improving visibility through the use of a tiny camera at the blade's end to provide a clear and magnified view of the larynx and surrounding structures during tracheal intubation. This enhanced visibility enables more accurate and efficient endotracheal tube positioning, reducing the risk of complications and thus improving patient safety. According to a systematic review published in the Cochrane Database of Systematic Reviews (CDSR) in 2022, video laryngoscopy reduces the rate of failed intubation, improves first-attempt success, and lowers the incidence of hypoxemic events and esophageal intubation compared to direct laryngoscopy in adults [15]. Interestingly, earlier systematic reviews, including one by the CDSR in 2016 and another by the British Journal of Anaesthesia in 2017 [16,17], did not find significant differences in these outcomes. These evolving conclusions reflect more than just a shift in visualization (from direct line-of-sight to enhanced video imaging); they also underscore the role of increased availability, improved training, and accumulated clinical experience with video laryngoscopy, which allowed its technical advantages to translate into tangible improvements in patient care [15,18,19].

The use of backup cameras and video laryngoscopes represents a significant advancement in risk mitigation and streamlining procedural efficacy. According to a 2016 IIHS study of crash data in 22 US states, backup cameras reduced back-over accidents by 16 to 40% [20], preventing potential accidents and reducing the risks associated with driving practices.

Similarly, video laryngoscopes enable more accurate and efficient tracheal intubation, decreasing the likelihood of adverse outcomes, patient risk [21], and the number of attempts to intubate in the operating room [18,19]. A Japanese study involving 270 patients in whom direct laryngoscopy failed after two attempts by a senior anesthesiologist reported a 99.3% success rate when video laryngoscopy was used as a rescue technique [22]. In trauma cases, where anatomical and situational challenges often complicate airway management, video laryngoscopy has also demonstrated higher first-attempt success rates as compared to direct laryngoscopy [23]. In ICU and operating room settings, the availability of video laryngoscopy is particularly valuable. In addition to facilitating intubation, video laryngoscopes enable the visualization of laryngeal structures and vocal cord movement, aiding in the diagnosis of conditions such as unilateral or bilateral vocal cord paralysis--an advantage not readily achievable with direct laryngoscopy.

Video laryngoscopes have established themselves as essential tools in managing complex airway scenarios, offering particular advantages in patients with an unstable cervical spine, maxillofacial trauma, or anticipated difficult airways. In such cases, minimizing cervical spine movement during intubation is critical, and video laryngoscopes enable better glottic visualization without requiring an alignment of oral, pharyngeal, and laryngeal axes. Several studies and clinical guidelines now recognize video laryngoscopes as a first-line approach or essential backup for scenarios where neck mobility is limited or the airway anatomy is distorted [24-26]. Furthermore, the improved first-pass success rate associated with video laryngoscopes contributes to reducing airway-related complications, such as hypoxia, esophageal intubation, and soft tissue trauma, in high-risk patients.

Education and training

Using tools such as backup cameras and video laryngoscopes in training and education is promising for helping beginners learn new skills, understand complex concepts, and develop self-assurance. Backup cameras enhance the learning curve for novice drivers, particularly with reversing and parking maneuvers. These technologies present visual representations that mimic the aerial illustrations frequently used in driving lessons, aiding comprehension of the vehicle's response to steering inputs and improving spatial awareness, which may be helpful for new drivers.

Similarly, video laryngoscopes are powerful pedagogical instruments in medical education, particularly in anesthesiology. They enable synchronous observation of the patient's airway by both the learner and the instructor, providing a clear, magnified view of anatomical structures that are often challenging to visualize during direct laryngoscopy. Indeed, compared with traditional direct laryngoscopy, video laryngoscopy provides a greater initial success rate for novices and medical students [27-30]. Some video laryngoscope models include a recording function that enables in-depth post-laryngoscopy and intubation analysis, which helps teach the airway management process and identify areas of improvement to prevent future mistakes [31]. A large, multicenter, randomized trial recently confirmed that video laryngoscopy provided a greater first-time intubation rate (85.1%) than direct laryngoscopy (70.8%) in EDs and intensive care units [19]. In this study, 91.5% of emergency medicine residents or critical care fellows performed orotracheal intubations, which may explain why both groups experienced similar rates of severe complications (21.4% and 20.9%, respectively), underscoring the significance of the video laryngoscope learning curve.

Limitations and misuse

Despite the significant improvements in the safety of backup cameras for driving and video laryngoscopes for tracheal intubation, it is crucial to recognize their limitations and potential for misuse. Although they enhance rear visibility, backup cameras are not substitutes for traditional environmental observation techniques such as checking backup and side mirrors or conducting manual over-the-shoulder checks. Drivers might erroneously rely too heavily on technology, disregarding these conventional safety checks. Furthermore, the effectiveness of backup cameras can be compromised under certain conditions, such as poor lighting, shade, heavy precipitation, or when the camera lens becomes obscured [11].

Analogously, while video laryngoscopes offer considerable benefits in visualizing the airway during intubation, they should not eclipse the fundamental skills of direct laryngoscopy. Overreliance on video laryngoscopy without proficiency in direct techniques may lead to serious complications, especially in scenarios where video devices are unavailable or malfunction. Although video laryngoscope-assisted intubation may appear intuitive, effective use is far from simple--it demands familiarity with a range of devices and deliberate training. These devices vary significantly in optical design and blade configuration. Some models follow the traditional Macintosh blade shape, others are hyperangulated, and some include a guiding channel integrated into the blade. As a result, each model presents unique characteristics and associated learning curves.

This complexity is particularly evident in the broader transition from direct to video-assisted techniques. Despite offering superior glottic visualization, video laryngoscopy introduces new challenges, including the paradox of ‘seeing but not intubating,’ where visualization does not always translate to procedural success without appropriate experience and manual dexterity. Studies have documented this mismatch between visualization and intubation success, particularly in patients with challenging airway anatomy or suboptimal positioning [32,33]. These challenges reinforce the continued importance of cultivating core airway management skills and underscore the need for device-specific techniques and dedicated training to navigate the unique geometry and mechanics of video laryngoscope systems.

It is also essential to keep in mind that video laryngoscopy-assisted intubation is not without its complications and has been associated with pharyngeal trauma. A retrospective, single-center study of 14,860 intubations revealed a 15-fold greater rate of oropharyngeal injury associated with video laryngoscopy than with direct laryngoscopy [34]. Decreased space between the blade and palate-pharyngeal wall, camera blind spots, frequent use of a rigid stylet, inadequate training, and inappropriate use may account for the high incidence of complications related to video laryngoscope-assisted intubations. Additionally, in certain clinical scenarios, such as facial trauma, head injury, or airway injuries accompanied by active bleeding, excessive secretions, or lens fogging, the use of video laryngoscopes may be limited for effective airway management.

Therefore, anesthesiologists must maintain both their direct and video laryngoscopy skills to choose the most effective approach for each patient. It is imperative to acknowledge that, in addition to devices, ensuring a secure airway relies on several factors, including careful planning, clinical knowledge, and years of experience.

Cost-effectiveness and economic impact

The economic evaluation conducted by the NHTSA on rear visibility technologies -ranging from ultrasonic sensors to rearview video systems and their combinations- demonstrates how integrating safety-focused innovations into daily practices can prevent harm and yield substantial financial and societal benefits [35]. These systems are estimated to prevent 95 to 112 fatalities and 7,072 to 8,374 injuries annually, particularly in backover accidents involving pedestrians and small children in vehicle blind zones, with the greatest advantages seen in larger vehicles like SUVs and pickups. Monetized benefits from these safety gains range from $446 million to $734 million per year, primarily due to lives saved, injuries avoided, and reduced long-term healthcare and litigation costs. While rearview video systems were the most expensive option, they delivered the highest net societal benefit when evaluated in terms of cost per life saved, outperforming more affordable but less effective alternatives like sensor-only systems. These estimates, presented in the U.S. Department of Transportation report ‘Rearview Video Systems’, highlight how technological interventions can justify their cost by preventing harm and reducing downstream costs [35].

Incorporating new technologies into clinical practice invariably raises important questions about cost and resource allocation. While video laryngoscopes entail higher initial acquisition and maintenance costs compared to traditional direct laryngoscopes, their broader impact on patient outcomes and healthcare system efficiency merits careful consideration. Although formal cost-effectiveness analyses directly comparing video laryngoscopy and direct laryngoscopy remain limited, several large trials and meta-analyses demonstrate that video laryngoscopy significantly reduces rates of failed intubation, multiple attempts, and complications such as esophageal intubation and aspiration [15,19,36,37]. These outcomes--strongly associated with increased healthcare costs--suggest that video laryngoscopy may offer economic value by minimizing adverse events and reducing resource utilization. From this perspective, the use of video laryngoscopes may yield indirect cost savings by improving patient safety, minimizing complications, and optimizing workflow efficiency, particularly in high-risk or time-sensitive clinical environments. Furthermore, the educational utility of video laryngoscopes--facilitating supervision, skill acquisition, and real-time feedback--may also contribute to cost-effective training, particularly in academic centers and teaching hospitals. Though more rigorous economic evaluations are needed, current clinical data suggest that video laryngoscopy offers a favorable value proposition when considering its broader implications for safety, training, and long-term outcomes.

Future perspectives

Emerging trends in backup camera technology are increasingly shaped by integration with the Internet of Things (IoT), enabling smarter and more connected vehicles. Modern rear-view systems now feature high-resolution imaging, real-time object recognition, and wide-angle lenses that enhance spatial awareness. With IoT connectivity, these cameras can communicate with cloud services and other onboard systems, such as collision avoidance, braking, and steering assist, to provide dynamic feedback and automated responses. Some models even transmit data to cloud platforms for continuous learning and predictive diagnostics. These advancements are pushing backup cameras beyond passive visibility aids into active safety and navigation tools, particularly beneficial for larger vehicles where traditional visibility remains limited [38].

With the rapid advancement of airway technologies, the evolution of video laryngoscopy is likely to continue in parallel with innovations in artificial intelligence (AI), robotics, and immersive training technologies. Recent developments highlight the integration of AI and robotic systems to assist in endotracheal intubation, promising enhanced precision, consistency, and decision-making support, especially in high-risk or complex cases [39]. This mirrors the growing interest in airway management technologies that integrate AI, connectivity, and real-time data feedback, suggesting that future advancements in laryngoscopy may similarly move toward intelligent, connected systems that go beyond imaging to provide predictive, assistive, and adaptive guidance.

Additionally, simulation-based education continues to expand the utility of video laryngoscopy in training environments. High-fidelity simulators, now often incorporating video laryngoscope views, allow learners to practice and refine intubation techniques, troubleshoot difficult airway scenarios, and build confidence without patient risk [40]. As simulation and virtual reality technologies mature, their integration with video laryngoscopy training is expected to further improve skill acquisition, retention, and team communication in airway management.

Ultimately, the next generation of airway tools will likely reflect a synthesis of technological innovation and human expertise, reinforcing rather than replacing core airway skills.

Conclusions

The backup camera and video laryngoscope share a common goal: using camera technology to provide an improved perspective for decision-making purposes. These methods benefit from the miniaturization and enhancement of image quality and visibility. Their systematic and widespread use yields increased safety, risk reduction, and educational advantages while sharing specific limitations and misuse traits. Although integrating a video laryngoscope has many benefits, it is crucial to understand that mastering fundamental skills, such as direct laryngoscopy, should not be overshadowed by technological advancements.

Finally, although video laryngoscopes and backup cameras may often be considered supplementary or nonessential tools, when appropriately indicated and applied, they can transform a potentially challenging, risky task into a straightforward, accurate, and safe procedure.
